# Supplementary material for: Intranasal oxytocin interacts with testosterone reactivity to modulate parochial altruism
Source: Commun Psychol. 2024 Mar 9;2:18. doi: 10.1038/s44271-024-00066-9 (PMC11332015; doi:10.1038/s44271-024-00066-9)
Supplement: Supplementary file 1 — Supplementary Information [file 44271_2024_66_MOESM1_ESM.docx]

**Supplementary Information for**

**Intranasal oxytocin interacts with testosterone reactivity to modulate parochial altruism**

**This PDF file includes:**

Supplementary Notes 1 to 6

Supplementary Figures 1 to 6

Supplementary Tables 1 to 8

**Supplementary Information**

**Supplementary Note 1: Sample size**

Due to a lack of studies that examined the interaction between oxytocin administration, endogenous testosterone reactivity, and sex, in the context of intergroup conflict, we could not locate a relevant effect size for estimating the sample size that will allow to achieve a statistical power of 0.8. This issue is compounded by the challenge of deriving power in two-level models^1^. A meta-analysis on the effect of intranasal oxytocin administration on face recognition, ingroup trust, and outgroup trust, included 21 studies with sample sizes ranging from 27 to 128 participants^2^. To our knowledge, the largest sample size of studies which applied intranasal oxytocin in the context of intergroup dynamics is 194^3^, with the exception of the Zhang et al. study^4^, which included 486 participants (however, for the statistical analysis of this study, each data unit represented the average of three participants, so the effective sample size is 162). Regarding testosterone reactivity, a meta-analysis reported on 16 studies that examined the association between testosterone reactivity and aggression, with sample sizes ranging from 30 to 201 participants^5^. Therefore, we decided to use a sample size of N = 204, which is one of the largest in the field.

**Supplementary Note 2: Sensitivity analysis**

To calculate the achieved statistical power, given our sample size (before and after excluding participants), we conducted Monte Carlo simulations at low, medium, and large effect sizes (1.68, 3.47, and 6.71, respectively^6^). For each effect size, we generated 1000 simulated datasets that were based on the characteristics of our sample (that is, the proportion of oxytocin and sex, and the mean and SD of testosterone reactivity), and on the parameters of the mixed logistic models that were conducted on the observed data. For each simulated dataset, we regressed investment on the two-way and three-way interactions between oxytocin, testosterone reactivity, and sex. The statistical power was calculated as the proportion of datasets, out of 1000, in which the odds ratios (OR) were significantly different from 1. For the two-way interactions, we could achieve very good power (power > .90) for estimating large and medium effect sizes (OR = 6.71 and 3.47, respectively), and low power (ranging from 0.32 to 0.52) for estimating small effect sizes (OR =1.68). For the three-way interaction, we could achieve reasonable power (.79 and .78 for sample sizes of 204 and 192, respectively) for estimating large effect size (OR = 6.71). However, we were able to achieve only low power (power < .5) for estimating medium and small effect sizes (OR = 3.47 and 1.68, respectively). Supplementary Table 1 and Supplementary Fig. 1 show a summary of the post hoc estimated statistical power by predictors.

**Supplementary Note 3: Secondary analyses – How do oxytocin and sex interact with different measures of testosterone reactivity in affect the likelihood to invest?**

In the main text, we report testosterone reactivity as the residual of T-levels when regressing T-levels following the task on T levels before the task. However, testosterone reactivity can also be measured as the ratio between T levels between two time-points or the absolute change in T levels between these points. As additional analyses to the regression models that are shown in the main text (Table 2), here, we show the results of the regressions with testosterone reactivity operationalized as a ratio (Supplementary Table 3) or as absolute change (Supplementary Table 4). As seen when comparing across tables, the main study findings (e.g., oxytocin × sex interaction and oxytocin × testosterone reactivity × sex interaction) are robust across all formulations.

**Supplementary Note 4: Secondary analyses – Intergroup coordination**

In the repeated intergroup chicken game, players can coordinate their investments within foursomes to maximize their payoffs (i.e., pure-strategy Nash equilibria). According to the Nash solution, only one player of a foursome invests in each round, and thus each player invests in 25.00% of the rounds^7^. However, in our study, the number of investors in a foursome was one only in 11.18% of the rounds, and the mean number of investors in each foursome was 2.47 (*SD* = 0.32) per round. The deviation from the Nash equilibria was also evident in the lag between two successive investments. The lag between two successive investments was four rounds, which is in accordance with the Pareto-optimal solution, was evident only in 2.97% of the cases. However, two investments in two successive rounds took place in 68.17% of the cases. These deviations from the Nash solution resulted in mean payoffs of 2.62 MU (*SD* = 2.12 MU) for a player per round, which is considerably lower than the expected payoffs of 4 MU under the Nash solution^7^. Finally, a higher number of investors per foursome was associated with lower payoffs of its members (*b* = -3.94, *SE* = 0.41, *p* < .001, Cohen’s *f*^2^ = 1.91, 95% confidence intervals (CI) = [-4.75, -3.12]; see Supplementary Fig. 2).

**Supplementary Note 5: Secondary analyses – Are changes in T levels dependent on time, oxytocin administration, and sex?**

To examine fluctuations in T levels during the experiment, we regressed T levels on time, oxytocin, and sex. Change in T levels from Time-1 to Time-2 were greater in male participants compared to female participants (*b* = 7.00, *SE* = 2.92, *p* = .017, Cohen’s *f*^2^ = 0.02, 95% CI = [1.25, 12.76]). However, changes in T levels from Time-2 to Time-3 and from Time-3 to Time-4 did not significantly differ between sexes (Time-2 to Time-3: *b* = 4.09, *SE* = 3.01, *p* = .176, Cohen’s *f*^2^ = 0.01, 95% CI = [-1.85, 10.03]); Time-3 to Time-4: *b* = 0.87, *SE* = 2.44, *p* = .722, Cohen’s *f*^2^ < 0.01, 95% CI = [-3.94, 5.68]). In female participants, changes in T levels were not significantly affected by time (Time-1 to Time-2: *b* = 1.12, *SE* = 1.18, *p* = .340, Cohen’s *f*^2^ = 0.01, 95% CI = [-1.18, 3.42]; Time-2 to Time-3: *b* = -0.11, *SE* = 0.95, *p* = .909, Cohen’s *f*^2^ < 0.01, 95% CI = [-1.96, 1.75]; Time-3 to Time-4: *b* = -1.28, *SE* = 0.89, *p* = .151, Cohen’s *f*^2^ = 0.02, 95% CI = [-3.04, 0.48]), or by the time × oxytocin interaction (Time-1 to Time-2: *b* = -1.31, *SE* = 2.35, *p* = .577, Cohen’s *f*^2^ < 0.01, 95% CI = [-5.91, 3.29]; Time-2 to Time-3: *b* = -1.07, *SE* = 1.89, *p* = .572, Cohen’s *f*^2^ < 0.01, 95% CI = [-4.78, 2.64]; Time-3 to Time-4: *b* = -0.46, *SE* = 1.86, *p* = .804, Cohen’s *f*^2^ < 0.01, 95% CI = -3.98, 3.06]). In male participants, however, T concentration levels increased from Time-1 (pre hormone administration) to Time-2 (25 m after hormone administration; *b* = 8.31, *SE* = 2.63, *p* = .002, Cohen’s *f*^2^ = 0.10, 95% CI = [3.15, 13.47]). There was no credible evidence that this rise in T levels was dependent on oxytocin (*b* = 0.05, *SE* = 5.27, *p* = .992, Cohen’s *f*^2^ < 0.01, 95% CI = [-10.27, 10.38]). For all other time points, changes in T levels were not significantly affected by time (Time-2 to Time-3: *b* = 3.93, *SE* = 2.85, *p* = .168, Cohen’s *f*^2^ = 0.02, 95% CI = [-1.66, 9.52]; Time-3 to Time-4: *b* = -0.43, *SE* = 2.56, *p* = .867, Cohen’s *f*^2^ < 0.01, 95% CI = [-4.80, 3.94]), or by the time × oxytocin interaction (Time-2 to Time-3: *b* = -6.51, *SE* = 5.67, *p* = .251, Cohen’s *f*^2^ = 0.01, 95% CI = [-17.62, 4.60]; Time-3 to Time-4: *b* = -4.74, *SE* = 3.89, *p* = .223, Cohen’s *f*^2^ = 0.01, 95% CI = [-3.96, 13.43]).

**Supplementary Note 6: Secondary analyses – Do oxytocin, testosterone reactivity, and sex interact to affect signaling?**

We conduct exploratory analyses to examine whether oxytocin, testosterone reactivity, and sex, influenced players' signals during the signaling period. Since each observation is nested also within round, we added rounds' ID as a random variable to the following models. Testosterone reactivity predicted the likelihood of players to signal ‘invest’ (OR = 2.12, *SE* = 0.20, *p* < .001, 95% CI = [1.76, 2.56]). Furthermore, male participants were more likely than female participants to signal ‘invest’ (OR = 1.31, *SE* = 0.14, *p* = .013, 95% CI = [1.06, 1.63]), and oxytocin decreased the likelihood of players to signal ‘invest’ (OR = 0.72, *SE* = 0.07, *p* = .001, 95% CI = [0.60, 0.88]). These effects persist even after controlling for situational cues (i.e., signals of other players in the prior seconds). The association between testosterone reactivity to the likelihood to signal 'invest' in male participants (OR = 2.57, *SE* = 0.35, *p* < .001, 95% CI = [1.96, 3.36]) was stronger compared to this association in female participants (OR = 1.78, *SE* = 0.28, *p* < .001, 95% CI = [1.31, 2.43]), resulting in a marginally significant effect of the two-way interaction between testosterone reactivity and sex on the likelihood of players to signal 'invest' (OR = 1.50, SE = 0.32, p = .061, 95% CI = [0.98, 2.28]). Other two-way interactions and the three-way interaction between oxytocin, testosterone reactivity, and sex, were not significant predictors of players' signals (oxytocin × testosterone reactivity: OR = 0.69, *SE* = 0.16, *p* = .113, 95% CI = [0.44, 1.09]; oxytocin × sex: OR = 0.99, *SE* = 0.24, *p* = .967, 95% CI = [0.61, 1.60]; oxytocin × testosterone reactivity × sex: OR = 1.91, *SE* = 0.86, *p* = .151, 95% CI = [0.79, 4.60]).

The interaction between oxytocin, testosterone reactivity, and sex did not significantly predict signals for any of the 29 s in the signaling period (all p's > .179; see Supplementary Table 5). A close look at the last seconds of the signaling period revealed an interesting pattern that led to the significant three-way interaction of the final decisions. In male participants, the interaction between oxytocin and testosterone reactivity predicted the likelihood of investing. However, it did not significantly predict signals at any of the former 29 s (all p's > .363; see Supplementary Table 6). Further analysis showed that the oxytocin × testosterone reactivity interaction on the likelihood to invest in male participants could be explained by a positive association between testosterone reactivity and the tendency of male participants under oxytocin to opt out in the last 3 s of the signaling period (OR = 8.26, SE = 6.71, *p* = .009, 95% CI = [1.68, 40.58]; see Supplementary Fig. 5). Neither oxytocin, testosterone reactivity, sex, nor their interactions significantly predicted the association between players' signals and final decisions (oxytocin: *b* = -0.60, *SE* = 0.63, *p* = .338, 95% CI = [-1.84, 0.63]; testosterone reactivity: *b* = 1.03, *SE* = 0.68, *p* = .129, 95% CI = [-0.30, 2.36]; sex: *b* = 0.17, *SE* = 0.70, *p* = .805, 95% CI = [-1.20, 1.55]; oxytocin × testosterone reactivity: *b* = -1.23, *SE* = 1.59, *p* = .440, 95% CI = [-4.34, 1.89]; oxytocin × sex: *b* = -1.10, *SE* = 1.34, *p* = .413, 95% CI = [-3.72, 1.53]; testosterone reactivity × sex: *b* = -0.31, *SE* = 1.49, *p* = .832, 95% CI = [-3.23, 2.60]; oxytocin × testosterone reactivity × sex: *b* = 1.95, *SE* = 2.82, *p* = .489, 95% CI = [-3.57, 7.47]).

**Supplementary References**

1. Arend, M. G. & Schäfer, T. Statistical power in two-level models: A tutorial based on Monte Carlo simulation. *Psychol. Methods* **24**, 1 (2019).

2. Van IJzendoorn, M. H. & Bakermans-Kranenburg, M. J. A sniff of trust: Meta-analysis of the effects of intranasal oxytocin administration on face recognition, trust to in-group, and trust to out-group. *Psychoneuroendocrinology* **37**, 438–443 (2012).

3. Ten Velden, F. S., Baas, M., Shalvi, S., Kret, M. E. & De Dreu, C. K. W. Oxytocin differentially modulates compromise and competitive approach but not withdrawal to antagonists from own vs. rivaling other groups. *Brain Res.* **1580**, 172–179 (2014).

4. Zhang, J. Y. Culture, Institutions and the Gender Gap in Competitive Inclination: Evidence from the Communist Experiment in China. *Econ. J.* **129**, 509–552 (2019).

5. Geniole, S. N. *et al.* Is testosterone linked to human aggression? A meta-analytic examination of the relationship between baseline, dynamic, and manipulated testosterone on human aggression. *Horm. Behav.* **123**, 104644 (2020).

6. Chen, H., Cohen, P. & Chen, S. How big is a big odds ratio? Interpreting the magnitudes of odds ratios in epidemiological studies. *Commun. Stat. Comput.* **39**, 860–864 (2010).

7. Bornstein, G., Budescu, D. & Zamir, S. Cooperation in intergroup, N-person, and two-person games of chicken. *J. Confl. Resolut.* **41**, 384–406 (1997).

Supplementary Figures and Tables

Plots show the estimated statistical power as a function of odds ratios and sample size. Power calculations were performed using Monte Carlo simulations. For each effect size, 1000 simulations were generated, and the hypothesis that the odds ratio of the interaction is different from 1 was tested. The solid vertical lines represent the observed odds ratio of each interaction for predicting the binary decision to invest. Dashed vertical lines represent small, medium, and large effect sizes.


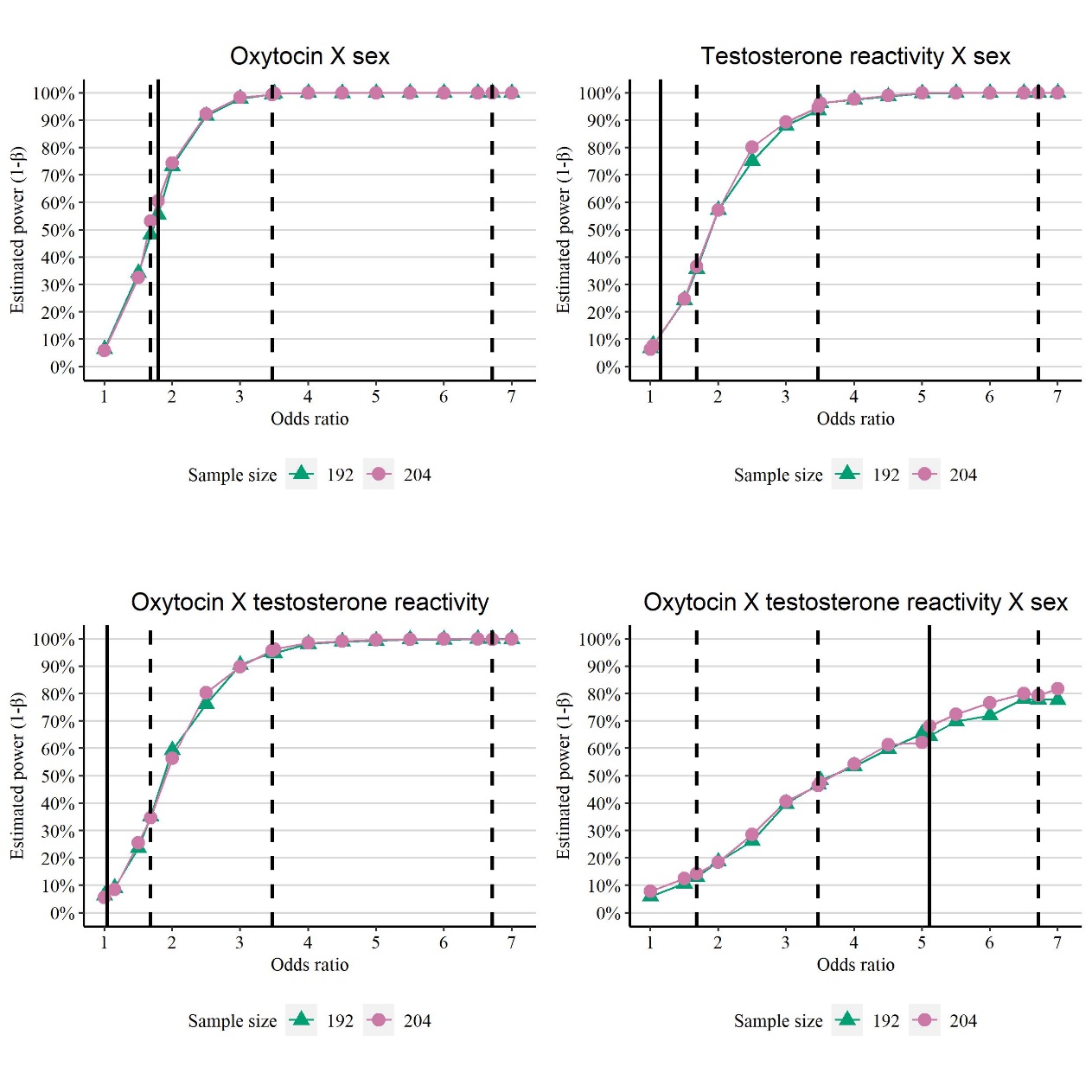


**Supplementary Fig. 1: Estimated statistical power as a function of effect size and sample size**

**Fig. S2.**

**Supplementary Fig. 2: Groups' payoffs by number of investors**

**Fig. S2.**


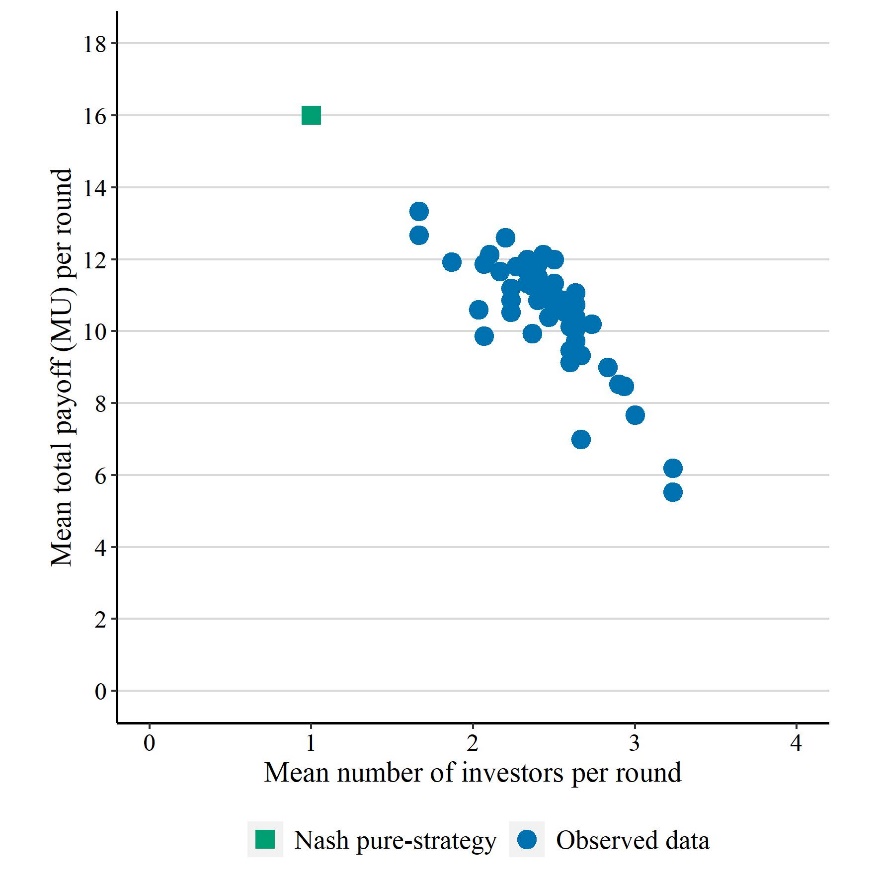


Plot of foursomes' mean total payoffs by mean number of investors per round. Each blue circle represents a foursome. Green square represents expected payoffs and number of investors for a foursome that would have completely followed the Nash pure-strategy.

Plot of mean total payoffs by mean number of investors per round. Each blue circle represents a foursome. Red circle represents expected payoffs and number of investors for a foursome that would have completely followed the Nash pure-strategy.

**Supplementary Fig. 3: Signal-change distributions by treatment and sex**


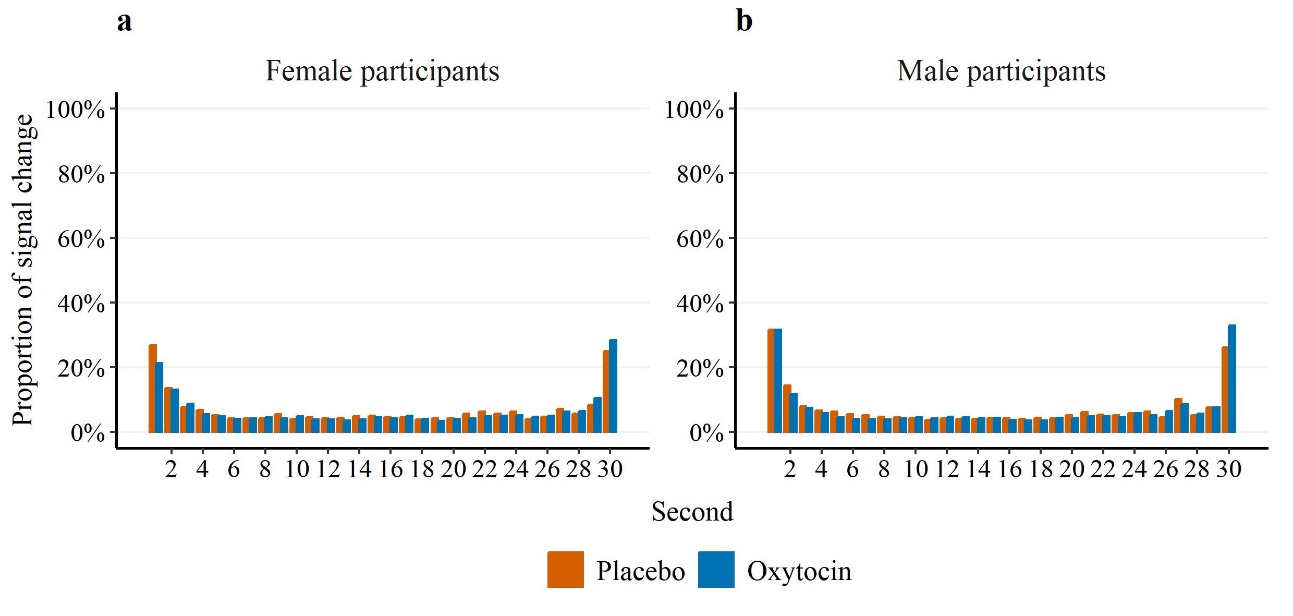


Plots show the proportion of players who changed their signal (from 'invest' to 'not invest' or vice versa) at each second of the signaling phase of the intergroup chicken game by treatment condition (oxytocin vs. placebo). Plots are shown separately for female participants (a) and male participants (b).

**Supplementary Fig. 4: 'Invest' signals' distributions by treatment and sex**

Plots show the proportion of players who signaled 'invest' et each second of the signaling phase of the intergroup chicken game by treatment condition (oxytocin vs. placebo). Plots are shown separately for female participants (a) and male participants (b). Panel a: n placebo = 45,900 observations (51 participants), n oxytocin = 45,900 (51 participants).


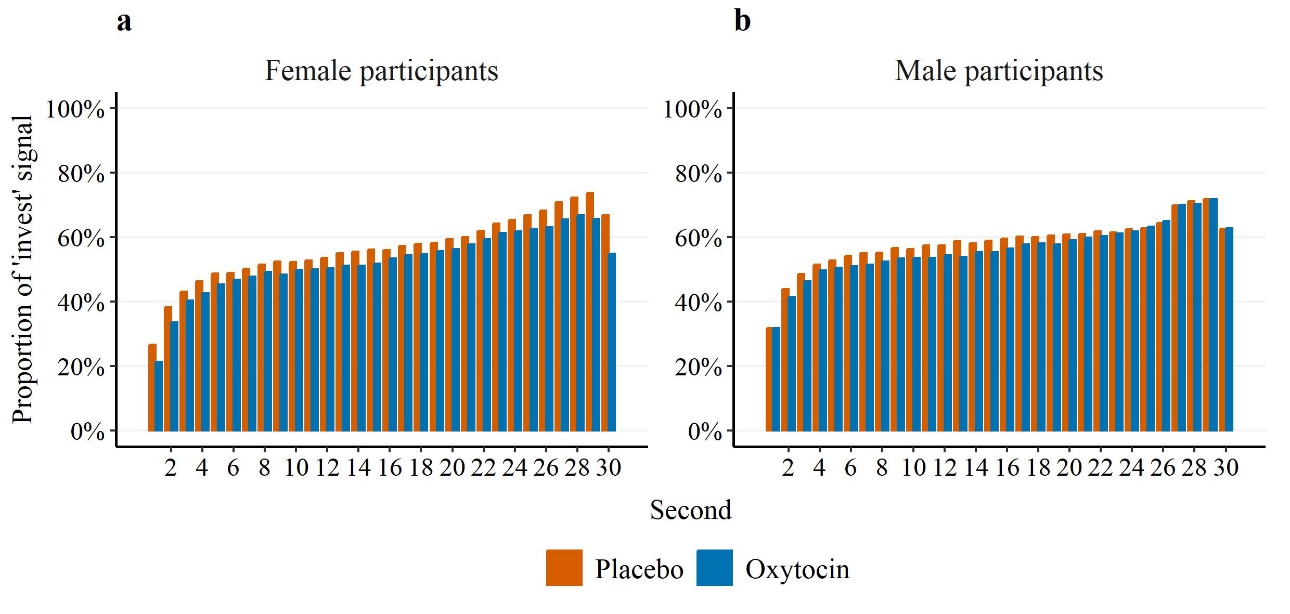


**Supplementary Fig. 5: Likelihood of male participants to opt-out during the last 3 s of the signaling period by treatment and testosterone reactivity**

**Fig. S3.**


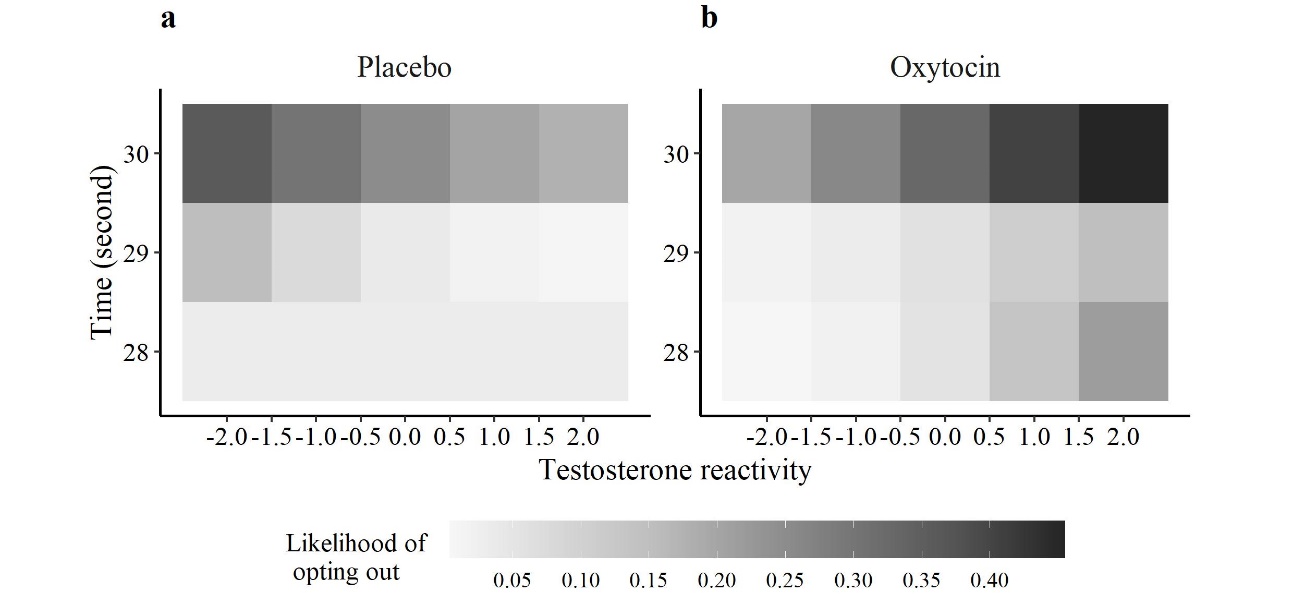


Plots show the relationship between testosterone reactivity during the intergroup chicken game, and the likelihood of players to change signal from 'invest' to 'not invest' during the last 3 s of the signaling period. Plots are shown separately for the placebo condition (a) and the oxytocin condition (b). Testosterone reactivity is based on residuals of predicting testosterone levels (standardized by sex) after the intergroup chicken game by testosterone levels (standardized by sex) before the game.

Plots by oxytocin of the relationship between testosterone (T) reactivity during the last 3 s of the Intergroup Chicken-Game signaling period, and the likelihood of players to change signal from 'invest' to 'not invest'. T-reactivity is based on residuals of predicting T levels (standardized by sex) after the Intergroup Chicken-Game by T levels (standardized by sex) before the game.

**Supplementary Fig. 6: Likelihood to invest by sex, testosterone reactivity, and oxytocin**

Relationship between testosterone reactivity during the intergroup chicken game, sex, and the likelihood of participants to invest their endowment. Plots are shown separately for the placebo condition (a) and the oxytocin condition (b). Testosterone reactivity is based on residuals of predicting testosterone levels (standardized by sex) after the intergroup chicken game by testosterone levels (standardized by sex) before the game. Panel a: n female participants = 1440 observations (48 participants), n male participants = 1410 (47 participants). Panel b: n female participants = 1410 observations (47 participants), n male participants = 1500 observations (50 participants).


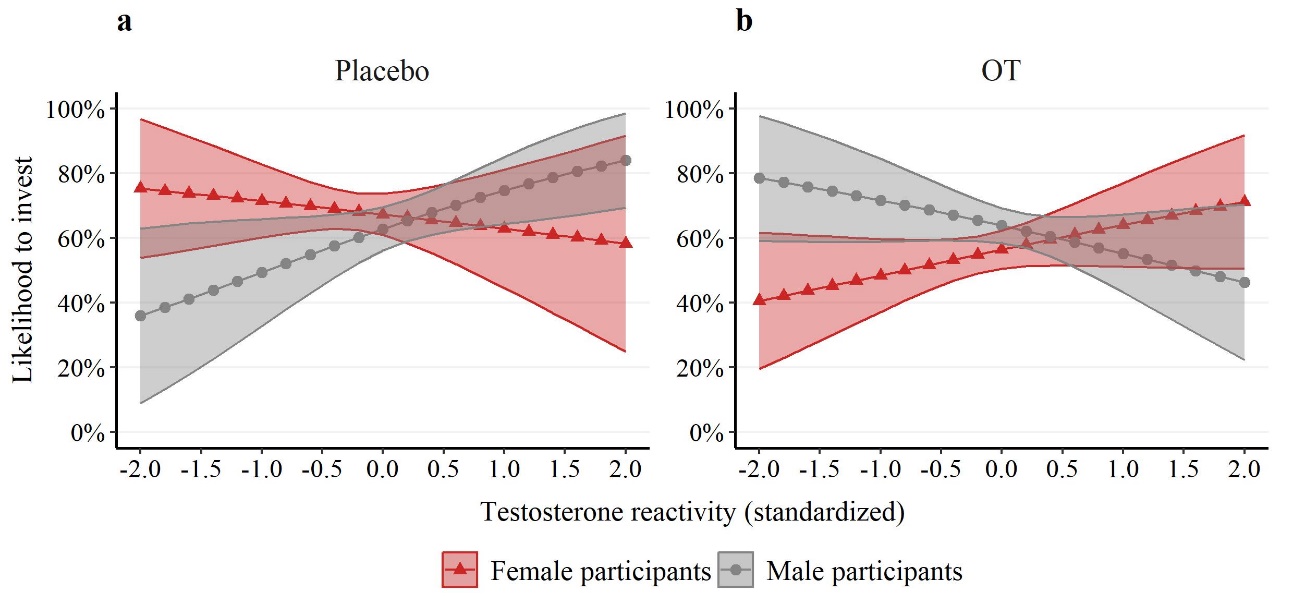


|  | Odds ratio (OR) | Estimated statistical power (N = 192) | Estimated statistical power (N = 204) |
| --- | --- | --- | --- |
| Oxytocin × sex | small (OR = 1.68) | .48 | .52 |
|  | medium (OR = 3.47) | > .99 | > .99 |
|  | large (OR = 6.71) | > .99 | > .99 |
|  | **observed data (OR = 1.79)** | **.56** | **.59** |
| Testosterone reactivity × sex | small (OR = 1.68) | .34 | .40 |
|  | medium (OR = 3.47) | .94 | .95 |
|  | large (OR = 6.71) | > .99 | > .99 |
|  | **observed data (OR = 1.15)** | **.09** | **.09** |
| Oxytocin × testosterone reactivity | small (OR = 1.68) | .32 | .36 |
|  | medium (OR = 3.47) | .97 | .95 |
|  | large (OR = 6.71) | > .99 | > .99 |
|  | **observed data (OR = 1.04)** | **.08** | **.07** |
| Oxytocin × testosterone reactivity × sex | small (OR = 1.68) | .13 | .14 |
|  | medium (OR = 3.47) | .47 | .46 |
|  | large (OR = 6.71) | .78 | .79 |
|  | **observed data (OR = 5.11)** | **.65** | **.68** |

The estimated statistical power for each observed interaction effect is marked in bold font.

**Supplementary Table 1: Estimated post-hoc statistical power by odds ratio and sample size**

*Estimated post-hoc statistical power by odds ratio*

| Model | (1) | (2) | (3) | (4) | (5) |
| --- | --- | --- | --- | --- | --- |
| Oxytocin | 0.77  [0.55, 1.10]  p = .148 | 0.58  [0.37, 0.89]  p = .012 | 0.59  [0.38, 0.90]  p = .016 | 0.63  [0.42, 0.95]  p = .026 | 0.67  [0.45, 0.99]  p = .042 |
| Male dummy | 1.06  [0.81, 1.38]  p = .677 | 0.79  [0.51, 1.21]  p = .281 | 0.80  [0.52, 1.23]  p = .308 | 0.80  [0.54, 1.20]  p = .284 | 0.81  [0.55, 1.20]  p = .288 |
| Testosterone reactivity ^a^ | 1.07  [0.77, 1.49]  p = .689 | 1.10  [0.63, 1.95]  p = .730 | 0.80  [0.40, 1.61]  p = .533 | 0.77  [0.41, 1.48]  p = .438 | 0.74  [0.39, 1.42]  p = .363 |
| Oxytocin × Male |  | 1.79  [1.07, 3.01]  p = .028 | 1.78  [1.07, 2.98]  p = .026 | 1.85  [1.12, 3.06]  p = .017 | 1.75  [1.05, 2.91]  p = .033 |
| Oxytocin × Testosterone reactivity |  | 0.87  [0.46, 1.65]  p = .668 | 1.80  [0.76, 4.26]  p = .181 | 1.98  [0.87, 4.48]  p = .103 | 2.13  [0.95, 4.77]  p = .067 |
| Male × Testosterone reactivity |  | 1.04  [0.55, 1.99]  p = .902 | 2.35  [0.93, 5.96]  p = .072 | 2.49  [1.03, 6.01]  p = .043 | 2.68  [1.12, 6.41]  p = .027 |
| Oxytocin × Male × Testosterone reactivity |  |  | 0.20  [0.06, 0.63]  p = .007 | 0.14  [0.04, 0.43]  p = .001 | 0.12  [0.04, 0.38]  p < .001 |
| Ingroup member's signal at the 29^th^ s |  |  |  | 3.62  [2.88, 4.55]  p < .001 | 3.70  [2.93, 4.66]  p < .001 |
| Outgroup members' signals at the 29^th^ s |  |  |  | 0.50  [0.43, 0.58]  p < .001 | 0.50  [0.43, 0.58]  p < .001 |
| Prior investments of ingroup member |  |  |  |  | 2.04  [1.32, 3.17]  p = .001 |
| Prior investments of outgroup members |  |  |  |  | 1.02  [0.76, 1.38]  p = .873 |
| Constant | 1.99  [1.48, 2.67]  p < .001 | 2.30  [1.64, 3.22]  p < .001 | 2.28  [1.63, 3.19]  p < .001 | 2.43  [1.71, 3.47]  p < .001 | 1.52  [0.86, 2.67]  p = .150 |
| Log Pseudolikelihood | -3583.90 | -3581.59 | -3578.71 | -3296.38 | -3155.00 |
| AIC | 7177.79 | 7179.19 | 7175.43 | 6614.76 | 6335.995 |
| BIC | 7211.09 | 7232.46 | 7235.36 | 6688.01 | 6422.12 |
| Number of Participants | 192 | 192 | 192 | 192 | 192 |
| Number of Observations | 5760 | 5760 | 5760 | 5760 | 5568 ^b^ |

Note: Factors contributing to the investment decision per round, were assessed via a repeated multilevel mixed effect logistic regression model. Male dummy = 1 if participant is male, 0 otherwise. Values in each cell represent odds ratios. Brackets contain 95% confidence intervals. Standard errors were clustered by dyads.

a – Testosterone reactivity was assessed by regressing testosterone levels (standardized by sex) following the completion of the intergroup chicken game onto testosterone levels (standardized by sex) before the game was initiated and saving the unstandardized residuals.

b – Model 5 does not include the first round since there are no prior investments.

**Supplementary Table 2: Multilevel logistic regression models of the players' likelihood to invest (standard errors clustered by dyads)**

Note: Factors contributing to the investment decision per round, were assessed via a repeated multilevel mixed effect logistic regression model. Male dummy = 1 if participant is male, 0 otherwise. Values in each cell represent odds ratios. Brackets contain 95% confidence intervals. Standard errors were clustered by foursomes.

a – Testosterone reactivity was assessed by calculating the ratio between testosterone levels (standardized by sex) following the completion of the intergroup chicken game and testosterone levels (standardized by sex) before the game was initiated.

b – Model 5 does not include the first round since there are no prior investments.

**Supplementary Table 3: Multilevel logistic regression models of the players' likelihood to invest (testosterone reactivity operationalized as the ratio between testosterone levels)**

| Model | (1) | (2) | (3) | (4) | (5) |
| --- | --- | --- | --- | --- | --- |
| Oxytocin | 0.77  [0.51, 1.19]  p = .240 | 1.34  [0.25, 7.10]  p = .730 | 0.24  [0.02, 3.05]  p = .274 | 0.21  [0.02, 2.55]  p = .222 | 0.18  [0.02, 2.10]  p = .172 |
| Male dummy | 1.06  [0.81, 1.39]  p = .671 | 0.83  [0.12, 5.73]  p = .853 | 0.16  [0.01, 2.11]  p = .164 | 0.17  [0.02, 1.87]  p = .145 | 0.15  [0.01, 1.51]  p = .107 |
| Testosterone reactivity ^a^ | 1.09  [0.40, 2.98]  p = .864 | 1.47  [0.31, 7.05]  p = .631 | 0.81  [0.12, 5.42]  p = .831 | 0.78  [0.13, 4.62]  p = .785 | 0.69  [0.12, 4.09]  p = .687 |
| Oxytocin × Male |  | 1.83  [1.09, 3.07]  p = .021 | 71.84  [2.54, 2031.78]  p = .012 | 144.57  [6.17, 3386.16]  p = .002 | 161.84  [7.25, 3610.66]  p = .001 |
| Oxytocin × Testosterone reactivity |  | 0.43  [0.08, 2.41]  p = .334 | 2.42  [0.15, 38.20]  p = .531 | 3.00  [0.21, 43.24]  p = .420 | 3.70  [0.27, 51.50]  p = .330 |
| Male × Testosterone reactivity |  | 0.95  [0.15, 6.19]  p = .957 | 4.89  [0.39, 61.70]  p = .219 | 4.81  [0.42, 54.99]  p = .207 | 5.49  [0.51, 58.98]  p = .159 |
| Oxytocin × Male × Testosterone reactivity |  |  | 0.03  [0.001, 0.72]  p = .031 | 0.01  [0.001, 0.31]  p = .007 | 0.01  [0.0005, 0.25]  p = .005 |
| Ingroup member's signal at the 29th s |  |  |  | 3.61  [2.85, 4.56]  p < .001 | 3.68  [2.89, 4.70]  p < .001 |
| Outgroup members' signals at the 29th s |  |  |  | 0.50  [0.43, 0.59]  p < .001 | 0.50  [0.42, 0.59]  p < .001 |
| Prior investments of ingroup member |  |  |  |  | 2.04  [1.40, 3.00]  p < .001 |
| Prior investments of outgroup members |  |  |  |  | 1.03  [0.76, 1.38]  p = .857 |
| Constant | 1.82  [0.70, 4.69]  p = .216 | 1.56  [0.35, 6.95]  p = .560 | 2.82  [0.47, 16.71]  p = .254 | 3.15  [0.57, 17.58]  p = .189 | 2.21  [0.34, 14.27]  p = .406 |
| Log Pseudolikelihood | -3583.95 | -3581.25 | -3579.18 | -3297.32 | -3156.30 |
| AIC | 7177.91 | 7178.51 | 7176.38 | 6616.64 | 6338.60 |
| BIC | 7211.20 | 7231.78 | 7236.305 | 6689.89 | 6424.72 |
| Number of Participants | 192 | 192 | 192 | 192 | 192 |
| Number of Observations | 5760 | 5760 | 5760 | 5760 | 5568 ^b^ |

| Model | (1) | (2) | (3) | (4) | (5) |
| --- | --- | --- | --- | --- | --- |
| Oxytocin | 0.77  [0.51, 1.19]  p = .240 | 0.57  [0.35, 0.93]  p = .025 | 0.59  [0.36, 0.97]  p = .037 | 0.64  [0.41, 0.99]  p = .044 | 0.67  [0.43, 1.04]  p = .076 |
| Male dummy | 1.06  [0.81, 1.39]  p = .659 | 0.79  [0.52, 1.21]  p = .284 | 0.82  [0.53, 1.25]  p = .352 | 0.82  [0.55, 1.23]  p = .338 | 0.83  [0.55, 1.23]  p = .352 |
| Testosterone reactivity ^a^ | 1.02  [0.73, 1.43]  p = .897 | 1.10  [0.67, 1.81]  p = .711 | 0.88  [0.48, 1.62]  p = .679 | 0.81  [0.46, 1.43]  p = .472 | 0.76  [0.43, 1.35]  p = .353 |
| Oxytocin × Male |  | 1.80  [1.09, 2.99]  p = .022 | 1.72  [1.05, 2.84]  p = .0.32 | 1.77  [1.06, 2.95]  p = .028 | 1.67  [0.98, 2.84]  p = .060 |
| Oxytocin × T-Reactivity |  | 0.82  [0.44, 1.51]  p = .522 | 1.51  [0.63, 3.59]  p = .355 | 1.72  [0.74, 4.00]  p = .205 | 1.83  [0.80, 4.18]  p = .153 |
| Male × Testosterone reactivity |  | 0.97  [0.52, 1.83]  p = .933 | 1.71  [0.70, 4.19]  p = .242 | 1.85  [0.77,4.46]  p = .169 | 2.00  [0.83, 4.84]  p = .124 |
| Oxytocin × Male × Testosterone reactivity |  |  | 0.28  [0.09, 0.88]  p = .030 | 0.19  [0.06, 0.61]  p = .005 | 0.18  [0.06, 0.57]  p = .003 |
| Ingroup member's signal at the 29^th^ s |  |  |  | 3.62  [2.86, 4.57]  p < .001 | 3.70  [2.89, 4.72]  p < .001 |
| Outgroup members' signals at the 29^th^ s |  |  |  | 0.50  [0.43, 0.59]  p < .001 | 0.50  [0.42, 0.59]  p < .001 |
| Prior investments of ingroup member |  |  |  |  | 2.06  [1.40, 3.02]  p < .001 |
| Prior investments of outgroup members |  |  |  |  | 1.03  [0.76, 1.38]  p = .867 |
| Constant | 1.99  [1.49, 2.65]  p < .001 | 2.30  [1.64, 3.23]  p < .001 | 2.28  [1.62, 3.21]  p < .001 | 2.44  [1.74, 3.43]  p < .001 | 1.51  [0.91, 2.51]  p = .114 |
| Log Pseudolikelihood | -3583.965 | -3581.53 | -3579.61 | -3297.48 | -3156.29 |
| AIC | 7177.93 | 7179.05 | 7177.225 | 6616.96 | 6338.58 |
| BIC | 7211.22 | 7232.32 | 7237.15 | 6690.21 | 6424.70 |
| Number of Participants | 192 | 192 | 192 | 192 | 192 |
| Number of Observations | 5760 | 5760 | 5760 | 5760 | 5568 ^b^ |

**Supplementary Table 4: Multilevel logistic regression models of the players' likelihood to invest (testosterone reactivity operationalized as the absolute difference between testosterone levels)**

Note: Factors contributing to the investment decision per round, were assessed via a repeated multilevel mixed effect logistic regression model. Male dummy = 1 if participant is male, 0 otherwise. Values in each cell represent odds ratios. Brackets contain 95% confidence intervals. Standard errors were clustered by foursomes.

a – Testosterone reactivity was assessed by calculating the absolute difference between testosterone levels (standardized by sex) following the completion of the intergroup chicken game and testosterone levels (standardized by sex) before the game was initiated.

b – Model 5 does not include the first round since there are no prior investments.

**Supplementary Table 5: Signals by the interaction effect between oxytocin, testosterone reactivity, and sex**

| **Second** | **Odds ratio** | **P. value** | **95% CI** |
| --- | --- | --- | --- |
| 1 | 1.11 | .936 | [0.09, 13.48] |
| 2 | 1.94 | .594 | [0.17, 22.43] |
| 3 | 2.86 | .368 | [0.29,28.32] |
| 4 | 2.64 | .396 | [0.28, 24.70] |
| 5 | 2.94 | .328 | [0.34, 25.39] |
| 6 | 2.55 | .386 | [0.31, 21.15] |
| 7 | 3.10 | .295 | [0.37, 25.75] |
| 8 | 3.72 | .183 | [0.54, 25.73] |
| 9 | 3.45 | .194 | [0.53, 22.28] |
| 10 | 2.64 | .305 | [0.41, 16.86] |
| 11 | 2.78 | .281 | [0.43, 17.88] |
| 12 | 3.15 | .211 | [0.52, 19.06] |
| 13 | 2.29 | .360 | [0.39, 13.57] |
| 14 | 2.65 | .278 | [0.46, 15.38] |
| 15 | 2.46 | .305 | [0.44, 13.79] |
| 16 | 2.68 | .257 | [0.49, 14.83] |
| 17 | 2.58 | .278 | [0.46, 14.34] |
| 18 | 2.18 | .365 | [0.40, 11.75] |
| 19 | 1.92 | .449 | [0.35, 10.40] |
| 20 | 2.25 | .369 | [0.38, 13.15] |
| 21 | 1.56 | .595 | [0.30, 8.15] |
| 22 | 1.23 | .804 | [0.24, 6.46] |
| 23 | 0.86 | .871 | [0.15, 4.98] |
| 24 | 1.06 | .945 | [0.20, 5.65] |
| 25 | 0.90 | .896 | [0.18, 4.55] |
| 26 | 0.74 | .723 | [0.14, 3.83] |
| 27 | 0.49 | .413 | [0.09, 2.68] |
| 28 | 0.48 | .371 | [0.09, 2.41] |
| 29 | 0.34 | .179 | [0.07, 1.64] |

Odds ratios of regressing players' signals on each second of the signaling period across 30 rounds of the intergroup chicken game on the interaction between oxytocin, testosterone reactivity, and sex. Each row includes 5760 observations (192 participants X 30 rounds).

**Supplementary Table 6: Signals of male participants by the interaction effect between oxytocin and testosterone reactivity**

| **Second** | **Odds ratio** | **P. value** | **95% CI** |
| --- | --- | --- | --- |
| 1 | 0.73 | .677 | [0.17, 3.22] |
| 2 | 0.99 | .990 | [0.22, 4.44] |
| 3 | 1.30 | .722 | [0.30, 5.64] |
| 4 | 1.29 | .732 | [0.29, 5.69] |
| 5 | 1.31 | .728 | [0.29, 5.93] |
| 6 | 1.26 | .757 | [0.29, 5.48] |
| 7 | 1.27 | .740 | [0.31, 5.29] |
| 8 | 1.70 | .447 | [0.43, 6.69] |
| 9 | 1.42 | .611 | [0.37, 5.51] |
| 10 | 1.33 | .676 | [0.35, 5.14] |
| 11 | 1.31 | .707 | [0.32, 5.31] |
| 12 | 1.42 | .603 | [0.38, 5.27] |
| 13 | 1.12 | .866 | [0.30, 4.22] |
| 14 | 1.29 | .711 | [0.33, 5.09] |
| 15 | 1.13 | .861 | [0.29, 4.31] |
| 16 | 1.24 | .754 | [0.33, 4.63] |
| 17 | 1.15 | .848 | [0.29, 4.57] |
| 18 | 1.02 | .981 | [0.28, 3.74] |
| 19 | 0.87 | .820 | [0.25, 3.01] |
| 20 | 1.04 | .955 | [0.30, 3.60] |
| 21 | 0.89 | .855 | [0.27, 2.95] |
| 22 | 0.88 | .840 | [0.26, 3.03] |
| 23 | 0.79 | .715 | [0.21, 2.87] |
| 24 | 1.04 | .950 | [0.31, 3.53] |
| 25 | 0.97 | .962 | [0.29, 3.23] |
| 26 | 1.05 | .934 | [0.30, 3.70] |
| 27 | 0.89 | .847 | [0.26, 3.03] |
| 28 | 0.76 | .641 | [0.24, 2.38] |
| 29 | 0.58 | .363 | [0.18, 1.86] |

Odds ratios of regressing male participants' signals on each second of the signaling period across 30 rounds of the intergroup chicken game on the interaction between oxytocin and testosterone reactivity. Each row includes 2910 observations (97 male participants X 30 rounds).

| Model | (1) | (2) | (3) | (4) |
| --- | --- | --- | --- | --- |
| Oxytocin | 0.77  [0.50, 1.18]  p = .236 | 0.58  [0.35, 0.94]  p = .026 | 0.59  [0.37, 0.95]  p = .029 | 0.63  [0.41, 0.97]  p = .034 |
| Male dummy | 1.06  [0.81, 1.39]  p = .679 | 0.79  [0.51, 1.22]  p = .285 | 0.80  [0.52, 1.23]  p = .311 | 0.80  [0.54, 1.20]  p = .283 |
| Testosterone reactivity ^a^ | 1.07  [0.76, 1.51]  p = .700 | 1.11  [0.63, 1.94]  p = .726 | 0.80  [0.40, 1.61]  p = .535 | 0.77  [0.40, 1.49]  p = .442 |
| Round Number | 1.01  [1.003, 1.019]  p = .009 | 1.01  [1.003, 1.019]  p = .009 | 1.01  [1.003, 1.019]  p = .009 | 0.01  [0.002, 1.020]  p = .018 |
| Oxytocin × Male |  | 1.79  [1.08, 2.98]  p = .025 | 1.79  [1.09, 2.93]  p = .022 | 1.85  [1.12, 3.07]  p = .017 |
| Oxytocin × Testosterone reactivity |  | 0.87  [0.47, 1.60]  p = .650 | 1.80  [0.72, 4.52]  p = .210 | 1.97  [0.80, 4.86]  p = .140 |
| Male × Testosterone reactivity |  | 1.04  [0.54, 2.02]  p = .904 | 2.35  [0.92, 6.01]  p = .074 | 2.49  [1.02, 6.04]  p = .044 |
| Oxytocin × Male × Testosterone reactivity |  |  | 0.20  [0.06, 0.64]  p = .007 | 0.14  [0.04, 0.43]  p = .001 |
| Ingroup member's signal at the 29th s |  |  |  | 3.54  [2.82, 4.45]  p < .001 |
| Outgroup members' signals at the 29th s |  |  |  | 0.49  [0.42, 0.58]  p < .001 |
| Constant | 1.69  [1.26, 2.28]  p = .001 | 1.95  [1.38, 2.78]  p < .001 | 1.94  [1.37, 2.74]  p < .001 | 2.15  [1.52, 3.03]  p < .001 |
| Log Pseudolikelihood | -3578.97 | -3576.67 | -3573.79 | -3291.86 |
| AIC | 7169.94 | 7171.34 | 7167.58 | 6607.71 |
| BIC | 7209.895 | 7231.265 | 7234.165 | 6687.615 |
| Number of Participants | 192 | 192 | 192 | 192 |
| Number of Observations | 5760 | 5760 | 5760 | 5760 |

**Supplementary Table 7: Multilevel logistic regression models of the players' likelihood to invest.**

Note: Factors contributing to the investment decision per round, were assessed via a repeated multilevel mixed effect logistic regression model. Male dummy = 1 if participant is male, 0 otherwise. Values in each cell represent odds ratios. Brackets contain 95% confidence intervals. Standard errors were clustered by foursomes.

a – Testosterone reactivity was assessed by regressing testosterone levels (standardized by sex) following the completion of the intergroup chicken game onto testosterone levels (standardized by sex) before the game was initiated and saving the unstandardized residuals.

| Model  **Supplementary Table 8: Multilevel logistic regression models of the players' likelihood to invest with data separated to earlier (1-20) and later (21-30) rounds** | (1) | | | (2) | | | (3) | | | (4) | | | (5) | | |  |
| --- | --- | --- | --- | --- | --- | --- | --- | --- | --- | --- | --- | --- | --- | --- | --- | --- |
| Round Number | | 1-20 | 21-30 | | 1-20 | 21-30 | | 1-20 | 21-30 | | 1-20 | 21-30 | | 1-20 | 21-30 | |
| Oxytocin | | 0.84  [0.56, 1.27]  p = .414 | 0.64  [0.36, 1.12]  p = .115 | | 0.62  [0.38, 1.01]  p = .055 | 0.49  [0.27, 0.88]  p = .017 | | 0.63  [0.39, 1.02]  p = .061 | 0.50  [0.29, 0.88]  p = .016 | | 0.68  [0.44, 1.03]  p = .068 | 0.54  [0.31, 0.92]  p = .023 | | 0.71  [0.46, 1.10]  p = .125 | 0.68  [0.43, 1.07]  p = .096 | |
| Male dummy | | 1.03  [0.82, 1.31]  p = .779 | 1.12  [0.75, 1.66]  p = .587 | | 0.76  [0.50, 1.14]  p = .184 | 0.86  [0.50, 1.50]  p = .601 | | 0.77  [0.51, 1.15]  p = .196 | 0.90  [0.52, 1.57]  p = .709 | | 0.75  [0.51, 1.10]  p = .136 | 0.95  [0.55, 1.62]  p = .841 | | 0.74  [0.50, 1.09]  p = .125 | 1.07  [0.66, 1.74]  p = .775 | |
| Testosterone reactivity ^a^ | | 1.04  [0.76, 1.43]  p = .790 | 1.13  [0.71, 1.82]  p = .607 | | 1.02  [0.61, 1.69]  p = .937 | 1.31  [0.59, 2.94]  p = .509 | | 0.79  [0.43, 1.45]  p = .456 | 0.77  [0.29, 2.08]  p = .610 | | 0.77  [0.45, 1.34]  p = .363 | 0.71  [0.28, 1.81]  p = .472 | | 0.72  [0.40, 1.28]  p = .266 | 0.81  [0.35, 1.84]  p = .610 | |
| Oxytocin × Male | |  |  | | 1.84  [1.07, 3.15]  p = .026 | 1.69  [0.93, 3.06]  p = .084 | | 1.84  [1.08, 3.11]  p = .024 | 1.65  [0.93, 2.93]  p = .086 | | 1.93  [1.14, 3.25]  p = .014 | 1.72  [0.91, 3.27]  p = .096 | | 1.82  [1.04, 3.18]  p = .035 | 1.41  [0.74, 2.68]  p = .296 | |
| Oxytocin × Testosterone reactivity | |  |  | | 1.04  [0.60, 1.79]  p = .893 | 0.55  [0.21, 1.42]  p = .219 | | 1.84  [0.84, 4.03]  p = .127 | 1.76  [0.43, 7.18]  p = .428 | | 1.86  [0.91, 3.80]  p = .089 | 2.39  [0.55, 10.36]  p = .246 | | 2.14  [1.04, 4.42]  p = .040 | 1.73  [0.50, 5.98]  p = .389 | |
| Male × Testosterone reactivity | |  |  | | 0.97  [0.51, 1.84]  p = .920 | 1.36  [0.53, 3.48]  p = .525 | | 1.82  [0.80, 4.17]  p = .156 | 5.56  [1.24, 24.92]  p = .025 | | 1.93  [0.88, 4.24]  p = .101 | 6.40  [1.61, 25.44]  p = .008 | | 2.15  [0.95, 4.84]  p = .065 | 5.43  [1.73, 17.00]  p = .004 | |
| Oxytocin × Male × Testosterone reactivity | |  |  | |  |  | | 0.28  [0.09, 0.85]  p = .025 | 0.07  [0.01, 0.44]  p = .004 | | 0.21  [0.08, 0.59]  p = .003 | 0.04  [0.01, 0.23]  p < .001 | | 0.18  [0.0, 0.51]  p = .001 | 0.05  [0.01, 0.25]  p < .001 | |
| Ingroup member's signal at the 29^th^ s | |  |  | |  |  | |  |  | | 3.31  [2.56, 4.29]  p < .001 | 5.21  [3.76, 7.23]  p < .001 | | 3.42  [2.60, 4.51]  p < .001 | 4.70  [3.47, 6.37]  p < .001 | |
| Outgroup members' signals at the 29^th^ s | |  |  | |  |  | |  |  | | 0.48  [0.40, 0.56]  p < .001 | 0.56  [0.43, 0.72]  p < .001 | | 0.47  [0.40, 0.56]  p < .001 | 0.59  [0.46, 0.76]  p < .001 | |
| Prior investments of ingroup member | |  |  | |  |  | |  |  | |  |  | | 1.67  [1.12, 2.50]  p = .012 | 12.30  [4.10, 36.86]  p < .001 | |
| Prior investments of outgroup members  Note: Factors contributing to the investment decision per round, were assessed via a repeated multilevel mixed effect logistic regression model. Male dummy = 1 if subject is male, 0 otherwise. Values in each cell represent odds ratios. Brackets contain 95% confidence intervals. Standard errors were clustered by dyads.  a – T-reactivity was assessed by regressing T levels (standardized by sex) following the completion of the Intergroup Chicken-Game onto T levels (standardized by sex) before the game was initiated and saving the unstandardized residuals.  b – Model 5 does not include the first round since there are no prior investments. | |  |  | |  |  | |  |  | |  |  | | 0.97  [0.73, 1.28]  p = .813 | 0.99  [0.50, 1.93]  p = .970 | |
| Constant | | 1.84  [1.41, 2.41]  p < .001 | 2.37  [1.62, 3.48]  p < .001 | | 2.14  [1.54, 2.97]  p < .001 | 2.71  [1.79, 4.11]  p < .001 | | 2.13  [1.54, 2.95]  p < .001 | 2.66  [1.77, 3.99]  p < .001 | | 2.64  [1.89, 3.70]  p < .001 | 1.80  [1.06, 3.05]  p = .028 | | 2.03  [1.12, 3.69]  p = .020 | 0.34  [0.10, 1.20]  p = .094 | |
| Log Pseudolikelihood | | -2452.62 | -1175.78 | | -2449.935 | -1173.98 | | -2448.03 | -1170.07 | | -2245.76 | -1073.77 | | -2110.50 | -1057.76 | |
| AIC | | 4915.24 | 2361.56 | | 4915.87 | 2363.95 | | 4914.06 | 2358.14 | | 4513.51 | 2169.57 | | 4247.00 | 2141.52 | |
| BIC | | 4946.51 | 2389.36 | | 4965.90 | 2408.435 | | 4970.34 | 2408.18 | | 4582.30 | 2230.73 | | 4327.63 | 2213.80 | |
| Number of Participants | | 192 | 192 | | 192 | 192 | | 192 | 192 | | 192 | 192 | | 192 | 192 | |
| Number of Observations | | 3840 | 1920 | | 3840 | 1920 | | 3840 | 1920 | | 3840 | 1920 | | 3468 ^b^ | 1920 | |

Note: Factors contributing to the investment decision per round, were assessed via a repeated multilevel mixed effect logistic regression model. Male dummy = 1 if participant is male, 0 otherwise. Values in each cell represent odds ratios. Brackets contain 95% confidence intervals. Standard errors were clustered by foursomes.

a – Testosterone reactivity was assessed by regressing testosterone levels (standardized by sex) following the completion of the intergroup chicken game onto testosterone levels (standardized by sex) before the game was initiated and saving the unstandardized residuals.

b – Model 5 does not include the first round since there are no prior investments.
