# Supplementary material for: Intranasal oxytocin interacts with testosterone reactivity to modulate parochial altruism
Source: Commun Psychol. 2024 Mar 9;2:18. doi: 10.1038/s44271-024-00066-9 (PMC11332015; doi:10.1038/s44271-024-00066-9)
Supplement: Supplementary file 3 — Description of Additional Supplementary Files [file 44271_2024_66_MOESM3_ESM.docx]

**Description of Additional Supplementary Files**

**File Name:** Supplementary Video 1

**Description:** On the left, a screen display for one round of the intergroup chicken game from an individual player's perspective. On the right, a visualization of player actions for the same round organized in matrix form. Rows and columns represent the number of investors for each team. Purple bubbles grow in size the longer the game is in that particular state. Visualization of the game was conducted using GridWare (Version 1.1; http://www.statespacegrids.org).
