# Supplementary material for: Intranasal oxytocin interacts with testosterone reactivity to modulate parochial altruism
Source: Commun Psychol. 2024 Mar 9;2:18. doi: 10.1038/s44271-024-00066-9 (PMC11332015; doi:10.1038/s44271-024-00066-9)
Supplement: Supplementary file 5 — Reporting Summary [file 44271_2024_66_MOESM5_ESM.pdf]

nature portfolio

Corresponding author(s): Dr Salomon Israel

Last updated by author(s): Feb 4, 2024

Reporting Summary

Nature Portfolio wishes to improve the reproducibility of the work that we publish. This form provides structure for consistency and transparency in reporting. For further information on Nature Portfolio policies, see our Editorial Policies and the Editorial Policy Checklist.

Statistics

For all statistical analyses, confirm that the following items are present in the figure legend, table legend, main text, or Methods section.

n/a	Confirmed	
☐	☒	The exact sample size (n) for each experimental group/condition, given as a discrete number and unit of measurement
☐	☒	A statement on whether measurements were taken from distinct samples or whether the same sample was measured repeatedly
☐	☒	The statistical test(s) used AND whether they are one- or two-sided<br>*Only common tests should be described solely by name; describe more complex techniques in the Methods section.*
☐	☒	A description of all covariates tested
☐	☒	A description of any assumptions or corrections, such as tests of normality and adjustment for multiple comparisons
☐	☒	A full description of the statistical parameters including central tendency (e.g. means) or other basic estimates (e.g. regression coefficient) AND variation (e.g. standard deviation) or associated estimates of uncertainty (e.g. confidence intervals)
☐	☒	For null hypothesis testing, the test statistic (e.g. F, t, r) with confidence intervals, effect sizes, degrees of freedom and P value noted<br>*Give P values as exact values whenever suitable.*
☒	☐	For Bayesian analysis, information on the choice of priors and Markov chain Monte Carlo settings
☐	☒	For hierarchical and complex designs, identification of the appropriate level for tests and full reporting of outcomes
☐	☒	Estimates of effect sizes (e.g. Cohen's d, Pearson's r), indicating how they were calculated

Our web collection on statistics for biologists contains articles on many of the points above.

Software and code

Policy information about availability of computer code

Data collection	No commercial software was used in data collection
Data analysis	Data were analyzed by R 4.1.1.1 and Stata 17.0

For manuscripts utilizing custom algorithms or software that are central to the research but not yet described in published literature, software must be made available to editors and reviewers. We strongly encourage code deposition in a community repository (e.g. GitHub). See the Nature Portfolio guidelines for submitting code & software for further information.

Data

Policy information about availability of data

All manuscripts must include a data availability statement. This statement should provide the following information, where applicable:

- Accession codes, unique identifiers, or web links for publicly available datasets
- A description of any restrictions on data availability
- For clinical datasets or third party data, please ensure that the statement adheres to our policy

Access to the experimental data that support the finding of this study can be obtained through the OSF repository at: https://osf.io/k78qp/?view_only=d50bf3b1d03b42019913c70abf19ebba

Human research participants

Policy information about studies involving human research participants and Sex and Gender in Research.

Reporting on sex and gender	The study included participants from both sexes (102 female participants). Participants' sex was determined based on self-report, and was considered in the study design. The source data includes information regarding participants' sex. All main analyses in the manuscript include participants' sex as one of the predictors.
Population characteristics	See Above.
Recruitment	Participants were recruited across multiple campus sites of the Hebrew University by the university's computerized recruitment system or by manual registration.
Ethics oversight	This study was approved by the Helsinki Committee of Hadassah Medical Center, Jerusalem (reference number: 0440-15-HMO).

Note that full information on the approval of the study protocol must also be provided in the manuscript.

Field-specific reporting

Please select the one below that is the best fit for your research. If you are not sure, read the appropriate sections before making your selection.

☐ Life sciences ☒ Behavioural & social sciences ☐ Ecological, evolutionary & environmental sciences

For a reference copy of the document with all sections, see nature.com/documents/nr-reporting-summary-flat.pdf

Behavioural & social sciences study design

All studies must disclose on these points even when the disclosure is negative.

Study description	Double-blind, placebo-controlled, between-subject design experiment.
Research sample	All participants were Hebrew University students (mean age = 24.47 y, SD = 2.40 y). All participants signed a written informed consent form before they participated in the study. Participants received 100 New Israeli Shekels (NIS; ~ 25$) or equivalent course credit for completing the study, and an additional fee (ranging from 0 to 21 NIS) based on their performance and decisions in the study.
Sampling strategy	Sample size was determined by aiming for equivalent sample size reported in studies which applied OT administration or measured T-reactivity in the context of aggressive behavior or intergroup dynamics (Supplementary Note 1 includes further details for the predetermination of the sample size).
Data collection	Data was collected via computer. 8 or 12 participants were present in each session and were were seated in front of computers at cubicles to prevent them to see each other screens. No one except from the experimenter and the participants was present in the room during the experiment. The researcher was blind to the conditions during data collection.
Timing	Data was collected between 23/3/2017-21/6/18
Data exclusions	No data were excluded from analysis.
Non-participation	No participants dropped out.
Randomization	participants were assigned to experimental conditions using stratified randomization, with sessions blocked for sex and treatment, such that each experimental session of 8/12 participants consisted of an equal number of OT and placebo, and an equal number of male and female participants.

Reporting for specific materials, systems and methods

We require information from authors about some types of materials, experimental systems and methods used in many studies. Here, indicate whether each material, system or method listed is relevant to your study. If you are not sure if a list item applies to your research, read the appropriate section before selecting a response.

Materials & experimental systems

n/a	Involved in the study
☒	☐ Antibodies
☒	☐ Eukaryotic cell lines
☒	☐ Palaeontology and archaeology
☒	☐ Animals and other organisms
☒	☐ Clinical data
☒	☐ Dual use research of concern

Methods

n/a	Involved in the study
☒	☐ ChIP-seq
☒	☐ Flow cytometry
☒	☐ MRI-based neuroimaging
